# The Uniform System of Accounts

**Published:** 1906-06-30

**Authors:** Francis H. Moore

**Affiliations:** Gen. Supt. and Sec. The Royal Infirmary, Liverpool.


### Editor's Letter-Box.

Our Correspondents are reminded that prolixity is a great bar to publication, and that brevity of style and conciseness of statement greatly facilitate early insertion.]

### THE UNIFORM SYSTEM OF ACCOUNTS.

Sir,—My letter of June 7, which you printed in last week's Hospital, was not intended for publication, or I should have refrained from reference to myself.

In view of the proposed revision of the "Uniform System of Accounts," I beg to make the following suggestions :—

Expenditure Tables.

*Provisions.*—Instead of combining "butter, cheese, and eggs"—as they are each important items of expenditure which it is desirable to check, it would be better to separate them. Cheese and bacon might go together. Tea is another item which, in some hospitals, the patients have to provide (a bad practice), which should not be obscured amongst "groceries."

*Alcohol.*—The main portion of "malt liquors" here is consumed by the staff and belongs to "provisions," as is cider, which is not a "malt" preparation. This objection also applies to "mineral waters," placed in "surgery" expenses.

*Surgery.*—The "Index" does not differentiate between "drugs" and "chemicals." What are the latter? "Ice" is not used solely for filling ice caps and checking hæmorrhages; it has an association with "provisions" to some extent.

*Domestic.*—The cost of "hardware" and "crockery" is now much mixed by the increased use of enamelled ware. Why not separate them? "Chandlery" is surely an obsolete term, since candles have gone out of use, and that commodity has little in common with cleanliness.

*Establishment Charges.*—What is meant by "house" in association with "medical" printing, etc.?

*Salaries and Wages.*—Why couple "medical" salaries (i.e., those of house surgeons, etc., which are not the invariable rule) with those of "dispensary"? "Other staff" in this sub-head seems unnecessary, and should not all salaries form part of "administration" expenses?

Classification Index.

Repairs of "furniture" and "fittings" I have always distinguished from those of what are usually called "fixtures," or part of the structure of the hospital, which latter have gone to "general repairs." "Boilers" are not "fixtures," but "fittings," therefore "repairs" is not the proper sub-head. "Brooms" and "brushes" are as much associated with "cleaning" as many of the articles allocated to that head; also "dusters," "sponge cloths," and "buckets."

"Candles" are here rightly placed to "lighting." Should not "bed," "diet," and "prescription" cards form part of "medical" printing, and "case books," "charts," etc.? "Gas governors" are as much "fittings" as gas burners, and cannot be considered as "lighting" any more than "boilers" are part of "fuel." A "guarantee premium" is surely a "salary." "Lactometer," why not a fitting"? "Laundry machinery" I place to "fittings" like any other machinery, and it is impossible to say how much of this belongs to any section. "Marking ink," part of linen, etc. "Methylated spirit," sometimes a large item, is omitted—used for heating bronchitis kettles, etc. "Grant to Pathologist Department," why not "medical salaries"? "Photographs of patients," now mainly Röntgen ray work—a sufficiently important item to be placed by itself, instead of in the limbo of "sundries," that pit of iniquity. "Rice" is as much "bread" as "oatmeal"—why not both to "grocery"? "Salaries of masseuses" would be more appropriately amongst "medical" than "nursing." Massage is a treatment, not service. "Salaries of laundresses" here go with "other wages." "Soap" for toilet purposes should be "grocery" "Operating table" seems to me an "instrument." 'Winding clocks," repairs of fittings.

I hope these suggestions may be of service. Yours truly,

Francis H. Moore,
Gen. Supt. and Sec.

The Royal Infirmary, Liverpool.
June 21, 1906.

